# Peripubertal GnRH and testosterone co-treatment leads to increased familiarity preferences in male sheep

**DOI:** 10.1016/j.psyneuen.2019.06.008

**Published:** 2019-10

**Authors:** D Hough, JE Robinson, M Bellingham, LM Fleming, M McLaughlin, K Jama, IRH Haraldsen, AK Solbakk, NP Evans

**Affiliations:** a College of Medical, Veterinary and Life Sciences, Institute of Biodiversity, Animal Health and Comparative Medicine, University of Glasgow, Glasgow G61 1QH, UK; b Department of Neuropsychiatry and Psychosomatic Medicine, Division of Surgery and Clinical Neuroscience, Oslo University Hospital – Rikshospitalet, 0027 Oslo, Norway; c College of Medical, Veterinary and Life Sciences, School of Veterinary Medicine, University of Glasgow, Glasgow G61 1QH, UK; d Department of Neurosurgery, Division of Surgery and Clinical Neuroscience, Oslo University Hospital – Rikshospitalet, 0027 Oslo, Norway; e Department of Psychology, University of Oslo, Pb 1094 Blindern, 0317 Oslo, Norway; f Department of Neuropsychology, Helgeland Hospital, 8607 Mosjøen, Norway

**Keywords:** Novelty, GnRH, Testosterone, Cognition, Motivation, Puberty

## Abstract

Chronic gonadotropin-releasing hormone agonist (GnRHa) treatment is effective for the medical suppression of the hypothalamic-pituitary-gonadal axis in situations like central precocious puberty and gender dysphoria. However, its administration during the peripubertal period could influence normal brain development and function because GnRH receptors are expressed in brain regions that regulate emotions, cognition, motivation and memory. This study used an ovine model to determine whether chronic peripubertal GnRHa-treatment affected the developmental shift from preference of familiarity to novelty. Experimental groups included Controls and GnRHa-treated rams. To differentiate between effects of altered GnRH signaling and those associated with the loss of sex steroids, a group was also included that received testosterone replacement as well as GnRHa (GnRHa + T). Preference for a novel versus familiar object was assessed during 5-min social isolation at 8, 28 and 46 weeks of age. Approach behavior was measured as interactions with and time spent near the objects, whereas avoidance behavior was measured by time spent in the entrance zone and attempts to escape the arena via the entry point. Emotional reactivity was measured by the number of vocalizations, escape attempts and urinations. As Control and GnRHa-treated rams aged, their approach behaviors showed a shift from preference for familiarity (8 weeks) to novelty (46 weeks). In contrast, relative to the Controls the GnRHa + T rams exhibited more approach behaviors towards both objects, at 28 and 46 weeks of age and preferred familiarity at 46 weeks of age. Vocalisation rate was increased in GnRHa treated rams in late puberty (28 weeks) compared to both Control and GnRHa + T rams but this effect was not seen in young adulthood (46 weeks). These results suggest that the specific suppression of testosterone during a developmental window in late puberty may reduce emotional reactivity and hamper learning a flexible adjustment to environmental change. The results also suggest that disruption of either endogenous testosterone signalling or a synergistic action between GnRH and testosterone signalling, may delay maturation of cognitive processes (e.g. information processing) that affects the motivation of rams to approach and avoid objects.

## Highlights

• Emotional reactivity was reduced in late puberty with testosterone suppression.
• GnRHa + testosterone led to greater approach and less avoidance behavior.
• Familiarity preferences increased post-puberty with GnRHa + testosterone treatment.
• Peripubertal GnRH + testosterone modulated motivation to approach/avoid objects.

## 1 Introduction

Gonadotropin-releasing hormone (GnRH) is, classically, thought of as a decapeptide that is synthesized in specialized hypothalamic neurons and transported via the hypophyseal portal vessels to the anterior pituitary gland, where it stimulates the release of the gonadotropins, luteinizing hormone (LH) and follicle stimulating hormone (FSH). As such, its importance in the regulation of pituitary-gonadal function has long been recognized. The presence of GnRH (types I and II) and GnRH receptors in other tissues (Hsueh and Schaeffer, 1985; Skinner et al., 2009), including brain regions that are not primarily involved in the regulation of reproductive function and behavior (Jennes et al., 1997; Wilson et al., 2006; Skinner et al., 2009; Schang et al., 2010), suggests that GnRH may have additional non-reproductive roles. In this regard, GnRH neurons extend to extra-hypothalamic brain regions such as the limbic system, which is important for the regulation of emotions, behaviors, motivations and memory. GnRH receptors are also expressed in such areas and GnRH has been shown to be able to stimulate extra-pituitary, neuronal, LH production (Wilson et al., 2006).

Within human and veterinary medicine, GnRH agonists (GnRHa) are used chronically to suppress activity within the hypothalamo-pituitary-gonadal axis (HPG). This is possible, as continuous activation of GnRH receptors leads to G protein receptor uncoupling, followed by internalization and recycling of receptors (Ferguson et al., 1996; Armstrong et al., 2011) and subsequent desensitization to the ligand. GnRHa-treatments are used in pediatric medicine to suppress pubertal changes in sex steroid hormones, in the treatment of gender dysphoria, central precocious puberty, idiopathic short stature, growth hormone deficiency, congenital adrenal hyperplasia, severe hypothyroidism and autism (Carel et al., 2009). While the physical and reproductive changes associated with GnRHa induced pubertal arrest have been described, additional effects such as changes in cognition, emotional control and motivated behaviors are not well characterized. However, it is imperative to investigate the effects of chronic GnRHa-treatment during the peripubertal period, as this represents a critical window of neuronal development, plasticity and programming (Berenbaum and Beltz, 2011) and therefore, any effects on brain function and behavior may be long lasting or even permanent.

Using an ovine model, we previously reported sex- and age-dependent effects of chronic peripubertal GnRHa-treatment on aspects of cognition, behavior and emotional reactivity (Wojniusz et al., 2011; Evans et al., 2012; Robinson et al., 2014; Hough et al., 2017). The interpretation of the results of these studies is complicated, as the outcomes may be influenced by the different testing conditions (Robinson et al., 2014) or associated with suppressed sex steroid concentrations. Nevertheless, a pattern is emerging that suggests some effects are mediated via chronic peripubertal GnRHa action on the cognitive processes that influence motivation. The results of earlier ovine studies indicate that chronic peripubertal GnRHa can result in alterations in avoidance behavior towards a novel object (Robinson et al., 2014), approach behavior towards a preferred food source (Wojniusz et al., 2011), emotional reactivity during social separation (Evans et al., 2012), and motivation to traverse a spatial maze to reunite with peers (Hough et al., 2017).

Novelty preference behavior can provide valuable insights to cognitive factors that influence motivation, as it reflects the outcome of judging associated risks against possible reward under different test conditions (Doyle et al., 2010; Ernst, 2014). In mammals, novelty preference behavior is sexually differentiated, with females typically exhibiting more negative emotions like fear/anxiety or avoidance to novel objects/situations compared to males. Novelty/familiarity preferences change with age in both sexes. At puberty changes in behavior that favors novelty and risk-taking become apparent, with a relative disregard for potential negative consequences, particularly in males (Spear, 2000; Ernst et al., 2009). It is likely that reproductive hormones have a regulatory role in such reactions and, therefore, these may be affected by blockade of the pubertal transition.

The aims of the present study were to 1) investigate the effects of chronic peripubertal GnRHa-treatment on novelty preference behavior as a measure of cognitive processes that influence motivation, and 2) differentiate between the effects of chronically disrupted peripubertal GnRH and gonadal steroid signaling in males before and after puberty.

## 2 Materials and methods

### 2.1 Animals and treatment

The research was conducted at the University of Glasgow Cochno Farm and Research Centre (55° 55′N) in accordance with Home Office Regulations (Project License: 60/4422). Animals were male Scottish Mule Texel crosses born between 23 March and 12 April 2013 (the mean age of animals on 30th March was 1 week). All animals came from single sex litters, to control for any effects of differences in the prenatal steroid environment associated with female siblings. Lambs were maintained with their dams until weaned at approximately 21 weeks of age. All sheep were grazed on pasture, except during behavioral trials, when indoor-housed sheep had *ad libitum* access to hay or silage and other nutritional supplements according to standard management practices.

At birth, lambs were randomly assigned to one of three treatment groups, with twins and triplets split across groups to minimize any maternal effects. The groups were: 1) untreated Control rams (Control, *n* = 60); 2) chronic GnRHa-treated rams (GnRHa, *n* = 55); and 3) chronic GnRHa-treated rams that also received testosterone replacement therapy (GnRHa + T, *n* = 24) to tease apart treatment effects associated with either GnRH or gonadal sex steroids. GnRHa-treatment (subcutaneous implant of goserelin acetate, Zoladex3.6 mg, kindly donated by Astra Zeneca, Macclesfield, UK) commenced at 8 weeks of age (pre-pubertal) and was administered every fourth week, until 1 year of age. To mimic the endogenous testosterone concentrations expected in the Controls, testosterone was replaced from 16 weeks of age until the end of the experiment via 2-weekly intramuscular injection of testosterone cypionate (A6960-000, Steraloids, Newport, USA) dissolved in vegetable oil as previously described (Wood et al., 1991). Based on the testosterone profiles published by Robinson et al. (2014), testosterone cypionate doses were: 16 weeks, 50 mg; 18–24 weeks, 120 mg; 26–30 weeks, 160 mg; 32–46 weeks, 240 mg; and 48 weeks, 136.4 mg.

### 2.2 Testes size and circulating testosterone concentrations

Every 4 weeks, from 8 weeks of age, on the day of GnRHa and testosterone cypionate administration, blood was collected, testes measured (length and circumference of scrotum in cm) and body weight (kg) recorded. Additional daily blood samples were collected from 10 GnRHa + T animals for 14 consecutive days in October (28–30 weeks of age) to characterize circulating testosterone concentrations in response to exogenous testosterone cypionate treatment (Supplemental Fig. 1). Blood samples (3 mL) were collected by jugular venipuncture and placed in heparin-treated tubes. After centrifugation at 5000 *g* for 15 min at 4 °C, plasma was harvested and stored at −20 °C. Testosterone concentrations were quantified by RIA, as previously described (Sheffield and O’Shaughnessy, 1989), following diethyl ether extraction from 50 μL plasma. Assay sensitivity averaged 0.02 ng/mL, and the intra- and inter-assay coefficients of variation were 8.1% and 5.9%, respectively. Assay of assay buffer/BSA supplemented with 1 ng/mL testosterone cypionate indicated that it did not cross react and was therefore undetectable with this RIA.

### 2.3 Assessment of novelty preference behavior

**Fig. 1:**
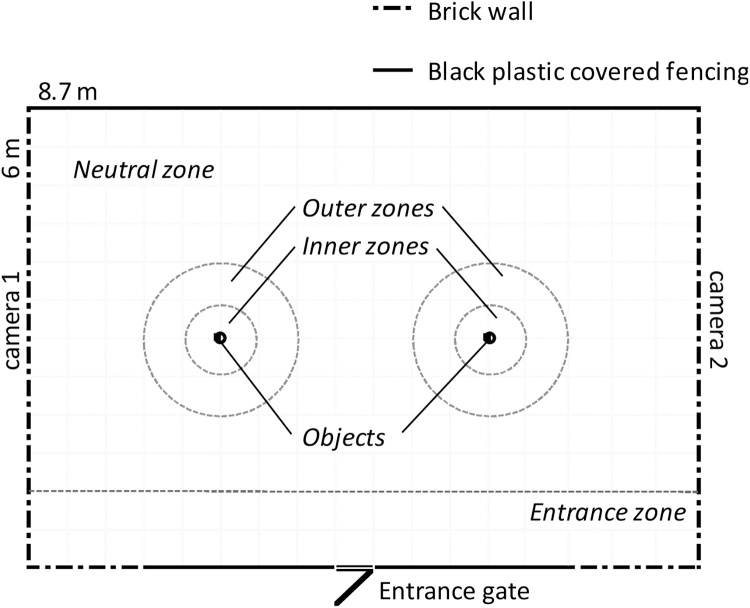
Design of the novelty preference arena. Zones were marked with spray-painted lines on the floor to assist in video analyses.

Novelty preference behavior was assessed over 2–4 days, at three developmental ages: 8 weeks (pre-pubertal, prior to GnRHa and testosterone treatment), 28 weeks (post-pubertal and during the breeding season) and 46 weeks (post-pubertal and during the non-breeding season). At each age, animals from the three groups were assessed in a random order (GnRHa + T animals were tested between 3–11 days post testosterone cypionate treatment (Supplemental Fig. 1). The indoor test arena (8.7 m × 6 m, Fig. 1) had concrete flooring and was bordered by either a concrete-brick wall or fencing covered with black plastic sheeting. A 1 m diameter ‘inner zone’ and a 2 m diameter ‘outer zone’ were marked on the floor surrounding the two test objects, to allow assessment of proximity to, and interest in an object (i.e. approach behavior). An ‘entrance zone’ was designated 0.5 m from the border with the gate. The remainder of the test arena floor area was designated a ‘neutral zone’. The behavioral test arena was fitted with two high-resolution day/night cameras (Cameras: KS-779VR, Eyeball 700 TV L, Vari-focal 2.8–12 mm; DVR: Guardian 4 G, K-DVR-4 G1, 500GB HDD; Digital Direct Security, Huntingdon, UK). To ensure familiarity with the test arena and the familiar object (two stacked red drinks crates), all animals had access to the test arena and the familiar object for approximately 24 h prior to the assessment. The novel objects were chosen to be of similar size and material, but different in shape and color from the familiar object. The novel object at 8 weeks of age was an orange traffic cone, at 28 weeks of age, a triangular yellow ‘wet floor’ sign, and at 46 weeks of age, a 5-gallon blue water bottle tied to a grey dustbin. After assessment of each animal, objects were sprayed with ethanol and wiped with paper towels to remove olfactory cues. Any feces or urine were also removed from the floor of the test arena and the area sprayed with ethanol. The placement of the novel and familiar objects on the right or left side of the area were alternated between each assessment.

For each assessment, one sheep was ushered through a gate into the test arena by an experienced handler. The sheep was allowed to explore the arena and its contents for 300 s, after which it was shepherded through the same gate with which it had entered. During each assessment, the time spent in the respective zones was quantified and the number of physical explorations of the novel and familiar objects (e.g. touching, licking, nudging, sniffing) recorded. Emotional reactivity was assessed by counting the number of vocalizations, escape attempts and urinations/defecations. An escape attempt was defined as a proactive effort to move through, over or under the border of the test arena. Revision of video footage was used to determine the time spent in each zone within the test arena. Presence in a zone was defined from when one foreleg was placed within that zone.

### 2.4 Statistical analyses

As statistical analysis indicated that body weight influenced testes size, the measure of testes size used in the analyses was calculated as (testes length × circumference)/body weight (cm^2^/kg). Time spent near an object was defined as the time an animal was within the inner and outer zones surrounding each object. Novelty preference was defined as the percentage of time spent near the novel object, relative to the total time spent near both objects, and the number of exploratory interactions with the novel object relative to the total number of object interactions. Statistical analyses were performed with R software (Version 3.2.5, © 2015 The R Foundation for Statistical Computing Platform) using the RStudio interface (Version 1.0.136, © 2009–2015 RStudio Inc.). All variables were analyzed with two-factorial ANOVA (Treatment × Age) using linear mixed-effects models with repeated measures (sheep identity specified as random factor) and the data distribution specified as Gaussian for continuous data (time spent in zones, % object preference, plasma testosterone and testes size, all of which were square root transformed for best fit based on the minimum Akaike information criterion for model selection) or Poisson for count data (number of interactions, urinations, escape attempts and vocalizations). To differentiate between the effects of GnRH and testosterone, respectively, two-tailed t-tests (or Wilcoxon signed-rank tests for non-parametric data) were used at each age (Control vs GnRHa, Control vs GnRHa + T, GnRHa vs GnRHa + T). All values presented are the mean and standard error of the mean of untransformed data. There were no between group differences, for any variable, prior to the start of the treatment at 8 weeks of age and, therefore, overall population means are presented in tables and figures. For all statistical tests an alpha-level of 0.05 was considered statistically significant and where alpha <0.09, trends are reported.

## 3 Results

### 3.1 Testes size and circulating testosterone concentrations

**Fig. 2:**
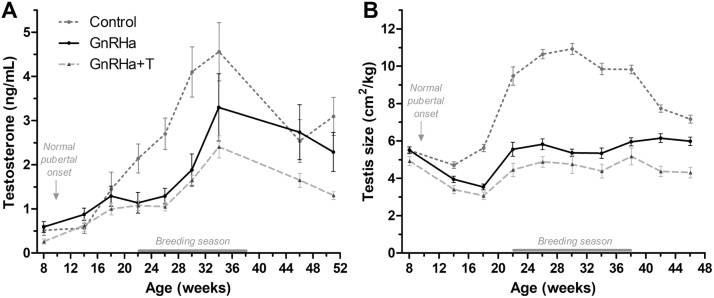
Changes in plasma testosterone (A) and testes size per body weight (B) of rams from pre- to post-puberty in Control, chronic peripubertal GnRHa-treated (GnRHa) and GnRHa-treated with testosterone cypionate replacement (GnRHa + T) rams. Values for GnRHa + T animals represent concentrations on day 14 after treatment with testosterone cypionate (Supplemental Fig. 1).

In Control rams, plasma testosterone concentrations and testes size increased from 12 weeks of age (Fig. 2) and reached a maximum between 26–36 weeks of age, after which both declined (Age *P* < 0.001). After 8 weeks of age, testes size was similar in both GnRHa-treated groups, but significantly (*P* < 0.001) lower compared to the Controls. While mean circulating testosterone concentrations increased in all groups, this increase was significantly delayed in both GnRHa-treated groups compared to Controls (Treatment × Age *P* = 0.004). Testosterone concentrations were significantly lower in GnRHa rams (*P* < 0.05) between 20–32 weeks of age. It should be noted that values in Fig. 2A for the GnRHa + T rams represent concentrations 14 days after testosterone cypionate administration (Supplemental Fig. 1).

### 3.2 Age effects

#### 3.2.1 Time allocation in test arena

At all ages, rams spent the most time in the neutral zone (Table 1). Overall, rams spent more time in the neutral zone at 28 weeks of age (55%), than at 8 (43%) and 46 (45%) weeks of age (Table 1, Table 2). At 8 weeks of age, rams spent more of the assessment time near both objects (33%) and less time in the entrance zone (24%), but this pattern was reversed at 28 (21% both objects, 24% entrance) and 46 (21% both objects, 34% entrance) weeks of age (Tables 1 and 2).

**Table 1:** Summary of the results for the time (in sec) spent in each zone of the arena and number of interactions with the objects during the 300-second testing period. Different superscript letters represent significant differences (*P* < 0.05) between treatment groups from t-tests at that age.

		8 wks	28 wks				46 wks			
		All animals *n* = 80	Control *n* = 42	GnRHa *n* = 39	GnRHa + T *n* = 23	All animals *n* = 104	Control *n* = 48	GnRHa *n* = 43	GnRHa + T *n* = 21	All Animals *n* = 102
*Zone times (sec)*										
Neutral		129.4 ± 6.1	171.0 ± 5.1	157.1 ± 5.4	166.3 ± 6.2	164.8 ± 3.2	132.2 ± 6.0	132.5 ± 6.3	145.0 ± 7.5	134.7 ± 3.8
Entrance		70.5 ± 4.5	71.7^ab^ ± 5.5	79.0^a^ ± 5.7	58.9^b^ ± 7.9	71.5 ± 3.6	107.4^a^ ± 6.3	105.4^ab^ ± 7.6	83.3^b^ ± 8.8	102.1 ± 4.4
Both objects		100.1 ± 6.1	57.3^a^ ± 3.5	64.9^ab^ ± 4.2	74.0^b^ ± 5.3	63.8 ± 2.5	60.4 ± 4.2	62.0 ± 4.2	71.7σ ± 4.8	63.1 ± 2.6
Familiar object		58.4 ± 5.1	31.1 ± 2.4	33.6 ± 3.0	40.0 ± 4.2	34.0 ± 1.8	28.2^a^ ± 2.4	30.3^a^ ± 2.5	43.1^b^ ± 4.4	31.8 ± 1.7
	Inner	26.6 ± 2.9	17.4 ± 1.9	18.0 ± 2.1	24.6 ± 3.2	19.2 ± 1.3	13.5^a^ ± 1.8	12.4^a^ ± 1.4	18.6^b^ ± 2.6	14.0 ± 1.1
	Outer	31.8 ± 2.7	13.7 ± 1.3	15.6 ± 1.6	15.4 ± 1.7	14.8 ± 0.9	14.7^a^ ± 1.1	17.9^a^ ± 1.6	24.5^b^ ± 2.7	17.8 ± 1.0
Novel object		41.7 ± 3.4	26.2 ± 2.1	31.3 ± 3.5	34.0σ ± 3.1	29.8 ± 1.7	32.2 ± 2.9	31.8 ± 2.5	28.6 ± 3.3	31.4 ± 1.7
	Inner	18.2 ± 1.8	15.6 ± 1.7	19.4 ± 2.7	18.8 ± 2.2	17.7 ± 1.3	16.5 ± 2.2	15.5 ± 1.5	15.1 ± 2.3	15.8 ± 1.2
	Outer	23.5 ± 2.2	10.6^a^ ± 1.0	11.8^a^ ± 1.6	15.2^b^ ± 1.7	12.1 ± 0.8	15.8 ± 1.3	16.3 ± 1.6	13.5 ± 1.7	15.5 ± 0.9
*Interactions (no.)*		6.5 ± 0.4	5.2^b^ ± 0.3	5.3^ab^ ± 0.4	6.4^a,^† ± 0.5	5.5 ± 0.2	5.0 ± 0.3	5.2 ± 0.4	6.1σ ± 0.6	5.3 ± 0.2
Familiar		3.6 ± 0.3	2.6^ab^ ± 0.2	2.3^b^ ± 0.3	3.3^a,^σ ± 0.4	2.8 ± 0.2	2.3^a^ ± 0.2	2.5^ab,^† ± 0.2	3.4^b^ ± 0.4	2.7 ± 0.1
Novel		2.9 ± 0.2	2.6 ± 0.2	2.9 ± 0.3	3.1 ± 0.4	2.6 ± 0.2	2.7 ± 0.2	2.7 ± 0.2	2.7 ± 0.4	2.6 ± 0.2

σ Control vs GnRHa + T *P* = 0.05-0.1.

† GnRHa vs GnRHa + T *P* = 0.05-0.1.

The amount of time animals spent near the novel and familiar objects (Table 1) significantly decreased with age (Table 2). In each case, the largest change was seen between 8 and 28 weeks of age, whereas the amount of time spent near these objects was similar between 28 and 46 weeks of age. Of the time spent near the objects, at 8 weeks of age, rams spent proportionately more time in the outer than in the inner zone of the objects (Table 1; Familiar object, 54% outer : 46% inner; Novel object, 56% outer : 44% inner). Interestingly, this pattern was reversed at 28 (Familiar object, 44% outer: 56% inner; Novel object, 41% outer: 59% inner), but not at 46 weeks of age (Familiar object, 56% outer: 44% inner; Novel object, 50% outer: 50% inner). The percentage of total time spent with the novel, relative to familiar object, did not change significantly (*P* = 0.335) with age as the novelty preferences across all animals assessed at 8, 28 and 46 weeks of age were 46 ± 2.8, 47 ± 1.8, and 50 ± 1.6%, respectively.

**Table 2:** Summary of P-values obtained from a two-factorial ANOVA (Treatment × Age) to assess the effects of treatment and age on the time spent in each zone of the arena and interactions with the objects during the 300-second test period. ***P < 0.001, *P < 0.05, ‘*P* < 0.1.

Arena area		Treatment	Age	Treatment x Age
*Zone times (sec)*				
Neutral		0.986	<0.001***	0.116
Entrance		0.082‘	<0.001***	0.282
Both objects		0.204	<0.001***	0.277
Familiar object		0.082‘	<0.001***	0.634
	Inner	0.105	<0.001***	0.865
	Outer	0.212	<0.001***	0.209
Novel object		0.913	0.008**	0.094‘
	Inner	0.841	0.673	0.386
	Outer	0.217	<0.001***	0.045*
*Interactions (no)*				
	Total	0.218	0.082‘	0.812
	Familiar	0.027*	<0.001***	0.596
	Novel	0.875	0.633	0.513

#### 3.2.2 Object explorations

**Fig. 3:**
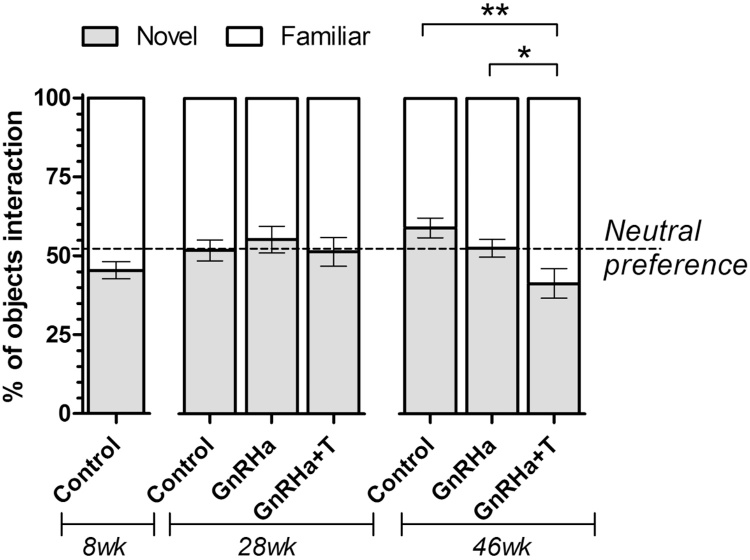
Percentage of interactions with the familiar and novel objects during the 300-second testing period at 8, 28 and 46 weeks of age. *P < 0.05, **P < 0.01.

The number of explorations of the familiar object decreased with age (Tables 1 & 2), whereas it remained unchanged for the novel object (Table 1, Table 2). Consequently, as rams aged the total number of interactions with the objects tended (Table 2) to decrease (Table 1), but the percentage of interactions with the novel object (Fig. 3) increased significantly from week 8 to weeks 28 and 46 (*P* < 0.05; 8 wk: 45 ± 2.7%, 28 wk: 52 ± 2.3%, 46 wk 51 ± 2.0%). There were no statistically significant difference in these parameters between weeks 28 and 46.

#### 3.2.3 Emotional reactivity

Overall, there was a significant decrease with age in the number of vocalizations (*P* < 0.001) and urinations (*P* < 0.05) during the testing period with the largest change seen between 8 and 28 weeks of age (Table 3). With age, rams were significantly (*P* < 0.001) more likely to make escape attempts (Table 3), with the largest increase seen between 28 and 46 weeks of age.

**Table 3:** Summary of results for the behavioral observations (number of events) during the novelty preference test. Superscript letters indicate results of t-tests with the same letters representing non-significant differences between treatment groups at that age.

Observation	8 wks	28 wks				46 wks			
	All Animals *n* = 77	Control *n* = 44	GnRHa *n* = 41	GnRHa + T *n* = 25	All Animals *n* = 110	Control *n* = 50	GnRHa *n* = 43	GnRHa + T *n* = 21	All Animals *n* = 114
Vocalizations	55.8 ± 2.2	18.7^ab^ ± 1.7	23.5^a^ ± 1.8	16.6^b^ ± 2.8	20.0 ± 1.2	9.7 ± 1.3	10.9 ± 1.7	12.3 ± 2.5	10.6 ± 1.0
Urination events	0.45 ± 0.08	0.32 ± 0.09	0.27 ± 0.07	0.32 ± 0.11	0.30 ± 0.05	0.26 ± 0.07	0.14 ± 0.06	0.19 ± 0.09	0.20 ± 0.04
Escape attempts	1.38 ± 0.29	2.73 ± 0.61	2.17 ± 0.66	1.36 ± 0.47	2.21 ± 0.36	8.02 ± 0.85	7.23 ± 0.97	6.33 ± 0.92	7.41 ± 0.55

### 3.3 Treatment effects

#### 3.3.1 Time spent in specific zones in the test arena

While there were no overall effects of treatment (Table 2) on time spent in each zone within the test arena (Table 1), there was a significant treatment by age interaction with regard to the time spent in the outer zone of the novel object. Specifically, the GnRHa + T group spent more time in this zone than the Control and GnRHa groups at 28, but not 46 weeks of age (Table 1).

Comparisons of treatment groups at 28 and 46 weeks of age indicated significant differences with regard to the time spent in different zones and explorations of the objects (Table 1). The GnRHa + T rams spent significantly less time in the entrance zone than the GnRHa rams at 28 weeks of age and the Control rams at 46 weeks of age (Table 1). The GnRHa + T group spent a significantly greater amount of time near both objects than the Control group at 28 weeks of age (*P* < 0.05) and a similar trend (*P* = 0.076) at 46 weeks of age. At 28 weeks of age the GnRHa+T rams spent more (*P* < 0.05) time in the outer zone of the novel object than the other groups, which was also reflected by a trend (*P* = 0.053) for GnRHa+T rams to spend more time near the novel object. However, at 46 weeks of age the GnRHa+T rams spent (*P* < 0.01) more time near the familiar object (Table 1) compared to the other groups. This effect was also noted in the time spent in the inner and outer zones of the familiar object (*P* < 0.05, Table 1). The percentage of time spent near the novel, relative to the familiar, object did not differ at 28 weeks of age (Control 45 ± 2.5%, GnRHa 47 ± 3.3%, GnRHa + T 48 ± 4.0%), but at 46 weeks of age the GnRHa + T group had lower (*P* < 0.01) novelty preferences (39±4.2%) than the Control (53±2.4%) and GnRHa (52 ± 2.4%) groups (Treatment *P* = 0.226, Treatment × Age *P* = 0.274).

#### 3.3.2 Object explorations

The GnRHa + T rams explored (Table 2) the familiar object more than the Control and GnRHa rams (Table 1). The number of exploratory interactions the GnRHa + T group had with the familiar object was similar at all ages (Table 1). Thus, there was a significant treatment effect (Treatment *P* = 0.021, Treatment × Age *P* = 0.333) on object interaction preference, whereby at 46 weeks of age GnRHa+T rams displayed a greater preference (Fig. 3) for exploratory interactions with the familiar object whereas the GnRHa and Control rams preferred interactions with the novel object.

#### 3.3.3 Emotional reactivity

Effects of treatment were limited to vocalizations at 28 weeks of age (Treatment × Age *P* < 0.001, Treatment *P* = 0.267). GnRHa rams vocalized more than GnRHa+T rams at 28 weeks of age and tended (*P* = 0.058) to vocalize more than Control rams (Table 3). There were no significant differences between treatment groups at 46 weeks of age.

## 4 Discussion

Novelty preference behavior provides valuable insight into cognitive processes that influence motivation as an individual balances the potential risk of the new, against the possible but unknown reward that may arise as a result of exploring an unfamiliar object. These cognitive processes can further be influenced by the conditions in which the novel item is encountered and the emotional state of the subject (Doyle et al., 2010; Ernst, 2014). Novelty preference in humans and animals is sexually dimorphic and changes during puberty. The results of this study show that chronic disruption of peripubertal GnRH signaling in rams, without testosterone replacement, neither altered the pre- to post-pubertal decline in approach behaviors towards familiar and novel objects nor the increase in preference for novelty, but was associated with increased vocalizations in late puberty (28 weeks of age). The replacement of testosterone ameliorated GnRHa-mediated differences in vocalization rates, but resulted in the disruption of age related changes in approach behavior where this did not decline with age, as in the other groups. These animals also showed a clear preference for familiarity in adulthood. As these animals received exogenous testosterone, the main physiological consequence of treatment was the disruption of GnRH signaling as opposed to both GnRH and testosterone signaling. Therefore the results suggest that both peripubertal GnRH and testosterone may modulate behavior and cognitive function during normal peripubertal development.

### 4.1 Testes size and circulating testosterone concentrations

Increased testes size and circulating testosterone concentrations after 14 weeks in Controls indicated that these animals had passed through the pubertal transition. GnRHa suppressed reproductive development, as in the GnRHa-treated groups changes in testis size were minimal. Despite this, there was a delayed increase in mean testosterone concentrations in GnRHa-treated rams, relative to the Controls, followed by a decrease after 34 weeks of age that would correspond with the end of the breeding season in the Controls. This result is similar to that reported for GnRHa-treated men where spermatogenesis i.e. reproductive potential was suppressed although circulating testosterone concentrations did not drop to hypogonadal levels (Bhasin et al., 1987). In the current experiment, the smallest testes:body weight ratio was seen in the GnRHa + T rams, which is consistent with other findings (Heber and Swerdloff, 1981; Bhasin et al., 1987) that GnRHa and exogenous testosterone act synergistically to inhibit gonadotropin secretion and thus testes size. Testosterone concentrations in the Controls declined towards the end of the experiment, which corresponds to the non-breeding season, whereas exogenous testosterone administration in the GnRHa + T rams was maintained. This discrepancy in circulating androgen concentrations, along with the loss of biological patterns of testosterone secretion in the GnRHa + T rams, may have exaggerated some effects at 46 weeks of age within this experimental paradigm.

### 4.2 Developmental shift from familiarity to novelty preference

With age, a shift was observed in the preference of Control rams from familiarity to novelty. A similar developmental transition towards novelty preference is reported in humans, where it can be observed from as early as 7 months of age (Shinskey and Munakata, 2010). This shift towards novelty preference has been ascribed to developmental changes in information-processing abilities regulated by the prefrontal cortex (Fagan et al., 2007) and reduced novelty preferences during infancy were associated with lower cognitive performance in adulthood (Fagan et al., 2007).

Interestingly, the effects on post pubertal novelty preference differed between the GnRHa and GnRHa + T rams. Rams that received both peripubertal GnRHa and testosterone replacement, had a lower post pubertal novelty preference. This could suggest that a loss of GnRH signaling is associated with disruption of the development of the neural circuits that regulate novelty/familiarity interactions that might negatively impact new learning and preference decision-making in adulthood. The same effect, however, was not seen in the rams in which both GnRH and testosterone signaling were suppressed. The lack of an effect in the GnRHa group could be explained if GnRHa-treatment modulated functions that are GnRH and testosterone-sensitive (i.e. act synergistically) or if the effects in the GnRHa + T rams are the result of maintained high concentrations of exogenous testosterone in this group relative to the Controls.

### 4.3 Object approach and avoidance behavior

While in the arena, the motivation of animals to approach objects was assessed by the time spent near the objects, which decreased from 8 to 28 weeks of age in Controls. This contrasts with an age dependent increase (between 28 to 46 weeks) in avoidance behavior, which for this study was reflected by the time spent in the entrance zone and the number of escape attempts, where animals expressed typical flight behaviors as they searched for their entry point and/or expressed a strong desire to return to their social peers. It is of note that many animals and humans share elements that regulate the functions of escape behaviors which take precedence over all other activities including social behavior, although some species or prey-predator differences may exist in safety and defense strategies (Dixon, 1998). If not engaged with the objects or expressing avoidance behaviors, it was possible for animals to spend time in the neutral zone where they did not display strong motivation to either approach or avoid the objects/escape the test arena, in the Controls this behavior was most pronounced at 28 weeks of age. This developmental shift from pre-puberty to young adulthood whereby approach behavior decreased, and avoidance behavior increased, is in line with human studies where these changes are attributed to temporal differences in the maturation of specific neural networks among the amygdala, ventral striatum and prefrontal cortex (Ernst et al., 2009; Ernst, 2014).

In the present study, the lack of an effect of GnRHa on the post-pubertal increase in avoidance and decrease in approach behaviors was similar to a previous study examining responses of male and female GnRHa treated (peripubertal) sheep to a novel object (Robinson et al., 2014). However, in the present study the replacement of testosterone significantly decreased avoidance behaviors, particularly the time spent in the entrance zone, and increased object approach behaviors. Thus, the peripubertal co-treatment with GnRHa and exogenous testosterone appears to have disrupted the maturational changes in cognitive processes that affect motivation, namely increased approach versus avoidance behavior towards objects in social isolation and/or decreased drive to flock. This observation is consistent with a former study with the same animals (Hough et al., 2017) where GnRHa + T rams exhibited reduced motivation to complete a spatial task to reunite with peers. In other studies, GnRHa-treated rams (no exogenous testosterone) also had reduced motivation to traverse a spatial maze (Wojniusz et al., 2013; Nuruddin et al., 2013c), and were more likely to engage in risk-taking behavior in a food-acquisition task (Wojniusz et al., 2011). The latter studies are also supported by data that indicated that peripubertal GnRHa-treatment is associated with an increase in amygdalae volume at 48 weeks of age (Nuruddin et al., 2013a). Collectively, these observations support our hypothesis that both peripubertal GnRH and testosterone signaling can affect maturational changes in selected cognitive processes that regulate motivation as a result of brain plasticity during adolescence.

### 4.4 Emotional reactivity

The decrease in the number of vocalizations and urinations with age in Control rams reflects the maturational change in emotional reactivity reported previously (Wosjniusz, et al., 2011; Evans et al., 2012; Robinson et al., 2014; Hough et al., 2017). In the current study, novelty preference was assessed while animals were in social isolation. Due to strong social grouping (Lyons et al., 1993), animals were probably tested in a state of heightened anxiety. Previous studies have shown that vocalization rates are markedly increased when sheep have no physical or visual contact with their peers (Lyons et al., 1993). Emotional state can result in judgement bias in sheep (Doyle et al., 2010) and as such, we would hypothesize that, within this experimental design, avoidance (i.e. ‘flight’, Dixon, 1998) would be favored over approach with animals spending more time in the entrance or neutral zones than close to objects.

As previously reported (Evans et al., 2012), vocalization rates were increased at 28 weeks of age in isolated GnRHa rams, relative to Controls, but this effect was lost with age. As this effect was not observed in the GnRHa + T rams, transient effects could be due to the ability of testosterone (endogenous in Controls or exogenous in GnRHa + T rams) to drive an aspect of emotional maturation that normally occurs early during the pubertal transition, but is not essential to reach maturity. Such a change in emotional reactivity could be related to the surges in emotional lability observed in humans at the advent of the pubertal transition and occur in association with developmental changes in the amygdala that continue into adulthood (Steinberg, 2008; Ernst, 2014).

Girls with central precocious puberty, who receive chronic GnRHa-treatment demonstrate increased emotional reactivity (Wojniusz et al., 2016) but lower resting heart rates and unchanged cognitive or psychosocial functioning. This suggests they process emotional stimuli differently. Our ovine studies have reported that chronic peripubertal GnRHa-treatment is associated with changes in amygdala volume in adulthood (Nuruddin et al., 2013a) although no changes in amygdala gene expression for selected markers of neuroplasticity or endocrine function were noted (Nuruddin et al., 2013b).

### 4.5 Potential mechanisms

The triadic model (Ernst, 2014) provides a framework to describe how age-dependent changes in motivated behaviors are regulated by temporal differences in the development and maturation of three neural systems, namely the prefrontal cortex, amygdala and ventral striatum. In this model, avoidance behavior is largely influenced by activity in neural hubs centered on the amygdala that process emotions and are particularly responsive to aversive stimuli (Ernst, 2014). Approach behavior is largely regulated by circuits centered on the ventral striatum, which is also implicated in controlling motivation through the dopaminergic mesolimbic system (Ernst, 2014). Finally, the activities of these two systems are coordinated by the prefrontal cortex, with its role in higher-order information processing and self-control (Ernst, 2014). The discordance in the development of these three neural systems in humans is believed to induce increased reward-seeking and risk taking behaviors from childhood to adolescence, due to early pubertal changes in the socio-emotional and motivational systems (Ernst, 2014; Steinberg, 2008). Conversely, the decline in risk-taking that is observed from adolescence to adulthood results from the later maturation of the prefrontal top-down driven control system that modulates approach and avoidance behaviors (Steinberg, 2008). In humans, the mid-adolescence period represents a window of particular vulnerability as the higher incidence of risk-taking can coincide with behaviors such as drug and alcohol misuse (Steinberg, 2008). In the present study, GnRHa and testosterone co-treatment reduced novelty preferences and directed decision-making towards more approach and less avoidance suggesting atypical maturation of the prefrontal cortex is likely.

It is possible that other neural networks may also be affected as a result of changes in the triadic model as a result of GnRHa and/or testosterone replacement for example those involved in memory, such as the amygdala-hippocampal network (Gawda and Szepietowska, 2016). Indeed, verbal cognition is facilitated, in part, through the amygdala-hippocampus network under regulation of the prefrontal cortex, as it is involved in initiating, searching and monitoring of memory. In this regard, it has been shown that people with low anxiety have better verbal fluency and exhibit increased activation of the right hemisphere, cerebellum and frontal gyri of the prefrontal cortex, all of which are thought to improve working memory and information processing (Gawda and Szepietowska, 2016). In the present study, vocalization rates, as potential indicators of anxiety, were reduced with the suppression of testosterone signaling. Similarly, vocalization rates during a spatial task were also reduced (Hough et al., 2017) by suppressing testosterone signaling. This reinforces the hypothesis that the amygdala-hippocampal network and prefrontal cortex function would be sensitive to changes in peripubertal testosterone concentrations and data from this ovine model indicates that peripubertal GnRH and/or testosterone can have differential effects in these neural regions.

The mechanism(s) through which GnRHa may affect the neural systems is poorly understood. Results that indicate persisting effects of elevated GnRH on cognitive function when sex steroids are replaced, argue against the hypothesis that these effects are secondary to the loss of sex steroids and this is supported by extensive studies in elderly men and women (Hogervorst, 2013). Even in such gerontological studies, it has been suggested that the age-related decline in some types of human cognition may be influenced by the associated increases in LH and/or GnRH in the absence of steroid negative feedback (Blair et al., 2015). Gordon et al. (1986) demonstrated that an acute injection of GnRH in healthy men (18–35 years of age) improved cognitive performance in a verbal fluency task, but no effects were observed with testosterone injections, which suggested a GnRH or LH specific effect. This observation is in line with studies showing that certain cognitive functions will decline with elevated GnRH, because chronic GnRH-treatment is expected to have the opposite effect of acute administration (Green et al., 2000). Using functional magnetic resonance imaging, Craig et al. (2007) reported that GnRHa-treatment over an 8-week period resulted in a reduction in verbal encoding (but not retrieval) that was associated with decreased hemodynamic activation in the left prefrontal cortex, anterior cingulate and medial frontal gyrus. Similarly, memory loss is a common side effect in men treated with GnRHa for metastatic prostate cancer (Flaig and Glode, 2008) and is associated with the decline in circulating concentrations of both androgens and estrogens (Salminen et al., 2004; Freedland et al., 2009). However, little is known about the cognitive effects associated with disrupted GnRH signaling in these patients.

## 5 Conclusion

The disruption of both peripubertal GnRH and testosterone signaling interfered with the normal maturational changes expected in certain aspects of cognitive processes that regulate motivation. There appears to be a window during late puberty or early adulthood where suppression of testosterone is associated with lower emotional reactivity, which will be of relevance for patients receiving GnRHa-treatment during this time. Increased motivation to approach objects and preferences for familiarity during young adulthood following peripubertal GnRHa and exogenous testosterone co-treatment may reflect reduced cognitive processes associated with the prefrontal cortex (e.g. reduced information processing or exaggerated approach as seen with adolescent risk-taking). The distinct difference between the GnRHa + T versus the GnRHa and Control groups may have resulted from loss of endogenous patterns of testosterone secretion, or a synergistic action between GnRH and testosterone signaling on selected cognitive processes during the peripubertal period. Imbalance of the fundamental (across species) motivational tendencies for approach and avoidance when encountering novel objects or situations may lead to reduced novelty seeking, and consequently hamper learning and flexible adjustment to environmental change. The findings may be of clinical relevance where chronic peripubertal GnRHa-treatment is administered with or without exogenous sex steroid treatments during this sensitive window as is the case in central precocious puberty and gender dysphoria.

Declarations of interest: none

## Declaration of Competing Interest

The authors declare no conflict of interest.
