# Supplementary material for: Peripubertal GnRH and testosterone co-treatment leads to increased familiarity preferences in male sheep
Source: Psychoneuroendocrinology. 2019 Oct;108:70–7. doi: 10.1016/j.psyneuen.2019.06.008 (PMC6712355; doi:10.1016/j.psyneuen.2019.06.008)
Supplement: Supplementary file 1 [file mmc1.pdf]

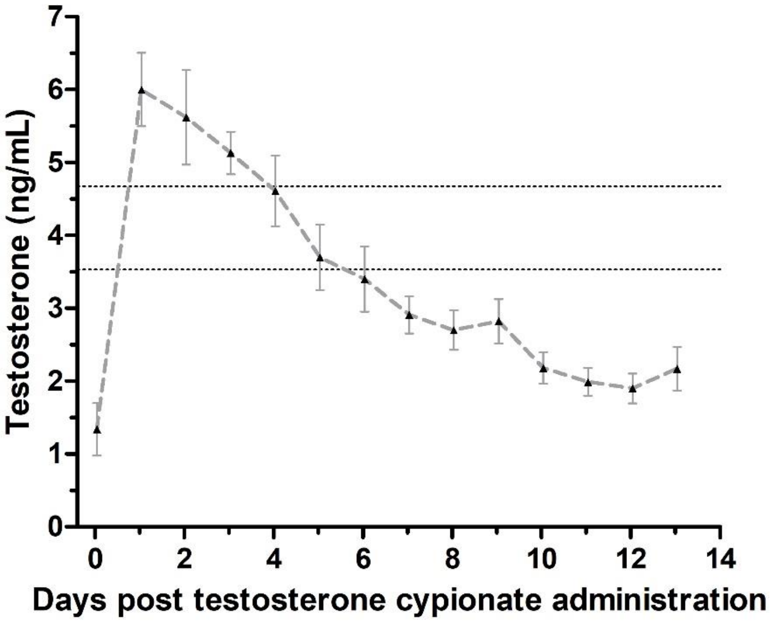

Supplementary Figure 1. Mean plasma testosterone concentration of 10 GnRH agonist treated rams immediately prior to (Day 0) and on the 13 days following a 160 mg testosterone cypionate i.m. injection at 28 weeks of age. The two horizontal gridlines represent the mean ± s.e.m. range of endogenous plasma testosterone concentrations observed for Control rams at 30 weeks of age (date corresponds to Day 14). All novel object tests, at all ages, were done between Days 3-11 post administration.
